# Supplementary material for: A systematic review assessing the potential for release of vector species from competition following insecticide-based population suppression of Anopheles species in Africa
Source: Parasit Vectors. 2021 Sep 8;14:462. doi: 10.1186/s13071-021-04975-0 (PMC8425169; doi:10.1186/s13071-021-04975-0)
Supplement: Supplementary file 1 — Additional file 1. Risk of bias analysis. Table S1. A summary of the results of risk of bias assessments undertaken on each individual study. Table S2. Studies with statistically significant changes in absolute densities of vector species. Table S3. Studies without statistically significant changes in absolute densities of vector species. Category ID studies dismissed from qualitative assessment in this review, as providing only weak evidence for competitive release or suggesting alternative explanations for changes in vector species composition. [file 13071_2021_4975_MOESM1_ESM.docx]

**Supplementary material for:**

**A systematic review assessing the potential for release of vector species from competition following insecticide-based population suppression of *Anopheles* species in Africa**

**ADDITIONAL FILE 1:** Risk of bias analysis. **Table S1.** A summary of the results of risk of bias assessments undertaken on each individual study. **Table S2.** Studies with statistically significant changes in absolute densities of vector species. **Table S3.** Studies without statistically significant changes in absolute densities of vector species. Category ID studies dismissed from qualitative assessment in this review, as providing only weak evidence for competitive release or suggesting alternative explanations for changes in vector species composition

**Risk of bias analysis**

A summary of the risk of bias analysis for each study was completed (Table S1). N=21 studies categorised as High or Very high for Overall risk of bias were eliminated from this investigation, leaving N=42 studies for further analyses. The most common risk of bias was from ‘Confounding factors’, where ‘Some concerns’ were found in N=30 studies. Factors here included any changes in the environment that might have affected the density of vectors other than the insecticide-based intervention itself, such as (1) changes in land use or farming practices such as irrigation in the vicinity of the study, (2) low bed net use (<60%) amongst study populations, in keeping with the Abuja Declaration from the African Summit on Roll Back Malaria [1], or (3) the presence or emergence of insecticide resistance. However, most of these studies (N=28) were assessed as ‘Low’ or ‘Some concerns’ for ‘Overall’ risk of bias, and were therefore included in subsequent analyses whilst taking any potential confounding factors into account. The most common reason (N=19) for studies to be assessed as having a High Overall risk of bias was because they were assigned to High risk of bias in the Missing data or data issues domain. Typically, such studies reported datasets that provided insufficient resolution of information to support unequivocal assessment against our inclusion/exclusion criteria, for example by providing density data graphically, rather than numerically.

**Table S1**. **A summary of the results of risk of bias assessments undertaken on each individual study.** Studies were assessed for risk of bias based on four domains, (1) ‘Missing data or data issues’, (2) ‘Lack of randomisation’, (3) ‘Confounding factors’ and (4) ‘Sample size/representativeness’, each of which contributed to an ‘Overall’ assessment of risk of bias. In order of ascending risk of bias, studies were classified as ‘Low’, ‘Some concerns’, ‘High’, and ‘Very high’. Studies where any domain was designated as High were classified as High for Overall risk of bias. Studies with two or more domains assessed as Some concern, but with the rest assigned as Low, were classified as Some concern for Overall risk of bias. Studies with three domains designated as Low, with the fourth domain assigned to Some concern, were classified as Low for Overall risk of bias.

| Study | Missing data or data issues | Lack of randomisation | Confounding factors | Sample size/  representativeness | Overall |
| --- | --- | --- | --- | --- | --- |
| Abong’o *et al* [2] | Low | Low | Low | Low | Low |
| Akono *et al.* [3] | Low | Low | Some concerns | Low | Low |
| Alegana *et al.* [4] | High | Low | Some concerns | Low | High |
| Antonio-Nkondji *et al.* [5] | Low | Some concerns | Some concerns | Low | Some concerns |
| Bamou *et al.* [6] | Some concerns | Low | Some concerns | Low | Some concerns |
| Bayoh *et al.* [7] | Low | Low | Low | Low | Low |
| Bekele *et al.* [8] | Some concerns | Low | Some concerns | Low | Some concerns |
| Bogh *et al.* [9] | Low | Low | Low | Low | Low |
| Bukhari *et al.* [10] | High | Low | Low | Low | High |
| Chanda *et al.* [11] | Low | Some concerns | No information | Low | Low |
| Coleman *et al.* [12] | High | Some concerns | Low | Low | High |
| Dabire *et al.* [13] | High | Low | Low | Low | High |
| Degefa *et al.* [14] | High | Low | Low | Low | High |
| Fontaine *et al.* [15] | Low | Some concerns | Low | Low | Low |
| Futami *et al.* [16] | Low | Low | Some concerns | Low | Low |
| Gillies and Smith [17] | Low | Some concerns | Low | Low | Low |
| Gimnig *et al.* [18] | Low | Low | Low | Low | Low |
| Gimnig *et al.* [19] | High | Low | Low | Low | High |
| Govella *et al.* [20] | Low | No information | Some concerns | Low | Low |
| Helinski *et al.* [21] | Some concerns | Low | Some concerns | Low | Some concerns |
| Kapesa *et al.* [22] | High | Low | Some concerns | Low | High |
| Kenea *et al.* [23] | High | Low | Low | Low | High |
| Kweka *et al.* [24] | Some concerns | Low | Some concerns | Low | Some concerns |
| Labbo *et al.* [25] | High | Some concerns | Low | Low | High |
| Lindblade *et al.* [26] | Low | Low | Some concerns | Low | Low |
| Lozano-Fuentes *et al.* [27] | Low | Low | Low | Low | Low |
| Mahande *et al.* [28] | High | Low | Low | Low | High |
| Majori *et al.* [29] | Low | Low | No information | Low | Low |
| Mbogo *et al.* [30] | Low | Low | Low | Some concerns | Low |
| McCann *et al.* [31] | Low | Low | Some concerns | Low | Low |
| Meyers *et al.* [32] | Low | No information | Some concerns | Low | Low |
| Meyrowitsch *et al.* [33] | Low | Low | High | Low | High |
| Mmbando *et al.* [34] | Low | Low | Some concerns | Low | Low |
| Molineaux *et al.* [35] | Low | No information | No information | Low | Low |
| Musiime *et al.* [36] | Low | No information | Some concerns | Low | Some concerns |
| Mutuku *et al.* [37] | High | Low | Low | Low | High |
| Mwangangi *et al.* [38] | High | No information | Some concerns | Low | High |
| Mwangangi *et al.* [39] | High | Low | Low | Low | High |
| Najera *et al.* [40] | High | Low | Low | Low | High |
| Njan Nloga *et al.* [41] | Low | Some concerns | Low | Low | Low |
| Njoroge *et al.* [42] | High | Low | Some concerns | Low | High |
| Odhiambo *et al.* [43] | Some concerns | Low | High | Low | High |
| Oloo *et al.* [44] | Low | Low | Low | Low | Low |
| Osse *et al.* [45] | Low | Some concerns | Low | Low | Low |
| Ouattara *et al.* [46] | High | Low | Some concerns | Low | High |
| Pant *et al.* [47] | Low | No information | Low | Low | Low |
| Pant *et al.* [48] | Low | No information | Low | Low | Low |
| Pinder *et al.* [49] | Low | Low | Low | Low | Low |
| Poche *et al.* [50] | Low | Low | Some concerns | Low | Low |
| Protopopoff *et al.* [51] | Low | Low | Low | Low | Low |
| Ratovonjato *et al.* [52] | High | Some concerns | Some concerns | Low | High |
| Russell *et al.* [53] | Low | Low | Low | Low | Low |
| Russell *et al.* [54] | Low | Low | Some concerns | Low | Low |
| Sharp *et al.* [55] | Low | No information | Some concerns | Low | Low |
| Smith and Draper [56] | Low | No information | Low | Low | Low |
| Smith [57] | High | Low | Low | High | High |
| Sougoufara *et al.* [58] | Some concerns | No information | Some concerns | Low | Some concerns |
| Sougoufara *et al.* [59] | Low | Low | Some concerns | Low | Low |
| Trape *et al.* [60] | Low | Low | Some concerns | Low | Low |
| Wragge *et al.* [61] | High | Low | Some concerns | Low | High |
| Zhou *et al.* [62] | Low | Low | Low | Low | Low |
| Zhou *et al.* [63] | Some concerns | Low | Some concerns | Low | Some concerns |
| Zhou *et al.* [64] | Low | Low | Some concerns | Low | Low |

**Table S2. Studies with statistically significant changes in absolute densities of vector species.** Statistical differences were identified from raw data analysis in each of the studies selected for detailed analysis of evidence for release from competition of vectors after suppression of *Anopheles* vector by insecticide-based intervention. *indicates statistically significant result.

| Study | Category of density change | Mosquito population density  decreased | Mosquito population density  increased | Mosquito  density  statistics | Malaria transmission | Malaria transmission statistics |
| --- | --- | --- | --- | --- | --- | --- |
| Akono *et al*. [3] | I | *n/a* | *An. coluzzi*, Cx. quinquefasciatus** | The biting rates were significantly higher after ITN distribution (*P* = 0.04, *P* = 0.03 respectively). | Increase | EIR difference was 0.83 and 3.06 infective bites per person per night before and after LLIN introduction respectively, however the difference between the two periods was not significant (*P* = 0.05). |
| Antonio Nkondji *et al.* [5] | D | *An. gambiae*, An. funestus*, An. nili*, An. moucheti** | *n/a* | All species reduced in individual densities.  Parity rates were significantly reduced (ꭓ^2^ = 55.0 ; *df* = 1; *P* < 0.0001). No other statistical analysis on densities provided. | Decrease* | The average infection rate of malaria vectors significantly decreased from 5.3% to 1.8% after bed net coverage (*P* < 0.0001). |
| Bayoh *et al.* [7] | ID | *An. gambiae* s.s.* | *An. arabiensis** | Density increased significantly with transect sampling position from Asembo to Seme in Poisson regression, and was observed for both sampling events (1^st^ transect sample: risk ratio = 1.28, 95% CI= 1.04-1.58, *P* = 0.006; 2^nd^ transect sample: risk ratio = 1.12, 95% CI = 1.02-1.24, *P* = 0.028). In the first transect sample, the (density-based) proportion of *An. gambiae* s.s. larvae relative to *An. arabiensis* was 16.7% in Asembo but 59% in Seme. In the second transect sample, the proportion of *An. gambiae* s.s. relative to *An. arabiensis* was 9.0% in Asembo and 56.6% in Seme.  The probability that an individual *An. gambiae* s.l. was identified as *An. gambiae* s.s. increased significantly (logistic regression) with transect sampling position from Asembo to Seme (1^st^ transect: risk ratio = 1.36, 95% CI = 1.09 – 1.68, *P* = 0.006; 2^nd^ transect: risk ratio = 1.77, 95% confidence interval = 1.49-2.10, *P* < 0.001). | n/a | n/a |
| Bogh *et al.* [9] | D | *An. gambiae* s.l*.*, Cx. quinquefasciatus*, An. funestus** | *n/a* | Comparison before and after intervention showed significant reduction in all *Anophelines* (*P* < 0.05)  A decrease in HBI reported for all species (*P* < 0.05). | n/a | n/a |
| Futami *et al.* [16] | D | *An. gambiae* s.l*.*, An. gambiae* s.s**, An. arabiensis** | *n/a* | *An. gambiae* s.l*.* and *An. gambiae* s.s reduced significantly by year (ꭓ^2^ = 940,78; *df* = 2, *P* < 0.001). *An. arabiensis* also reduced significantly (*P* < 0.001). | n/a | n/a |
| Gillies and Smith [17] | ID | *An. funestus** | *An. rivulorum** | There was a statistically significant difference between mosquito densities as determined by one-way ANOVA (*F*_(3,70)_ = 5.057, *P* = 0.003. A LSD post hoc comparison revealed that the mean number of *An. funestus* collected before intervention was significantly greater than that of *An. rivulorum* (*P* = 0.017)*,* and that the mean number of *An funestus* collected after intervention was significantly lesser in number compared with *An. rivulorum* (*P* = 0.013)*.* | n/a | n/a |
| Gimnig *et al.* [18] | D | *An. gambiae* s.l*.*, An. funestus* s.l*.** | *n/a* | Indoor resting densities of *An. gambiae* s.l*.* and *An. funestus* s.l*.* were significantly lower in the intervention houses (58.5%; *P* = 0.010 and 94.5%; *P* = 0.001). | Decrease* | Sporozoite infection rate significantly reduced in *An. gambiae* s.l*.* in intervention areas  EIR estimated to be reduced by 90%. |
| Govella *et al.* [20] | D | *An. gambiae* s.l**, Cx.* spp.*** | *n/a* | *An. gambiae* s.l*.* GLMM analysis (ρ = 1 -Relative Rate (RR) 95% CI = 99.2 [93.9-99%])  *Cx.* spp. GLMM analysis (ρ = 1 - (RR) 95% CI = 91.4 [91-92%]). | n/a | n/a |
| Helinski *et al.* [21] | ID | *An. funestus** | *An. gambiae* s.l*.** | Average number of *An. gambiae* s.l*.* and *An. funestus* s.l*.* per house per night collected by light traps, significance taken from error bars on graph. | Decrease* | Vector infectivity was 3.2% at baseline and 1.8% three years post-distribution (*P* = 0.001). |
| Kweka *et al.* [24] | D | *An. gambiae* s.l*.*, Cx.* spp.* | *n/a* | A reduction in mean mosquito densities, data taken from graphs, significance denoted by error bars. | n/a | n/a |
| Lindblade *et al.* [26] | D | *Anopheles*, An. arabiensis*, An. funestus**, *Culicines** | *n/a* | Using proportional density data:  The proportion of *An. arabiensis* differed significantly between treatment arm (48.8%) and non-treatment arm (22.6%), *P* < 0.0001)  *An. funestus* comprised a significantly lower percentage of the total number of *Anophelines* in the intervention arm (11.6%) compared with non-intervention arm (44%; *P* = 0.0001)  The indoor resting density of all *Anopheles* was significantly lower in the intervention arm (*P* = 0.001)  The number of *Culicines* per house was significantly lower in the intervention arm (*P* = 0.001). | Decrease* | Intervention zone had a lower percentage of anophelines with sporozoites than the non-intervention zone (*P* < 0.001). |
| Mbogo *et al.* [30] | D | *An. gambiae* s.l*.*, An. funestus** | *n/a* | Reduction in ranks of mean zonal densities between before and after intervention for both species (Mann-Whitney U test, *P* = 0.0001). | Decrease | Decrease was not significant |
| McCann *et al.* [31] | D | *An. gambiae* s.s**, An arabiensis** | *n/a* | Houses with an LLIN had more female *An. gambiae* s.s and *An. arabiensis* than houses where some people (rate ratios, 95% CI 0.87,0.85-0.89; 0.84, 0.82-0.86; 0.38, 0.37-0.40) or everyone used an LLIN (RR, 95% CI 0.49, 0.48-0.50; 0.39, 0.39-0.40; 0.60,0.58-0.61). | n/a | n/a |
| Meyers *et al.* [32] | D | *An. gambiae*, An. melas*, An. coluzzii** | *n/a* | Decrease in densities for all mosquitoes across all sites, with *An. gambiae* s.l*.* noted as significant through abundance ratios. Significance for *An. melas* and *An. coluzzii* noted through error bars on graph. | n/a | n/a |
| Mmbando *et al.* [34] *(indoor data observations only)* | ID | *An. gambiae*, Culex* spp.*** | *An. funestus*, Mansonia* spp. | All P values represent incidence Rate Ratio (IRR) % protection reductions of number of host-seeking mosquitoes attempting to bite volunteers outdoors in local households with push-pull, versus controls:  *An. gambiae* complex, 0.26, *P*<0.001  *An. funestus* group -0.48, *P*<0.05  *Mansonia* spp. -0.1, *P*>0.05  *Culex* spp. 0.23, *P*<0.01. | n/a | n/a |
| Molineaux *et al.* [35] | D | *An. gambiae* s.l*.*, An. funestus*, An. pharoensis** | *n/a* | Positive association between prespraying NBC/PSC ratio for *An. gambiae* s.l*, An. funestus* and *An. pharoensis,* and the residual density under propoxur. | n/a | n/a |
| Musiime *et al.* [36] | D | *An. gambiae* s.s**, An arabiensis** | *n/a* | Adjusted Incidence Rate Ratio of 0.07 difference between pre and post intervention, in total human biting rate. Proportions are provided for *An. gambiae* s.s and *An. arabiensis.* | Decrease | EIR reduced from 129 to 0. |
| Oloo *et al.* [44] | ID | *An. funestus** | *An. gambiae* s.l*.** | Mean house densities/vector/month, with a difference between before and after intervention using data from graphs – *An. funestus* showed decrease whereas *An. gambiae* s.l. showed increase. | Decrease | EIR was reduced by 72% in the intervention village. |
| Osse *et al.* [45] | D | *An. gambiae*, Mansonia* spp.*. | *n/a* | Significant reduction in blood feeding and rate of both species, and parity rate for *An. gambiae.* | Decrease | Reductions of over 70% of EIR, no statistical analysis provided. |
| Pinder *et al.* [49] | D | *An. gambiae* s.l*.*,*  *An. gambiae* s.s**, An. arabiensis*, Cx.* spp. | *n/a* | *An. gambiae* s.l*.* and sibling species varied by year (before and after intervention), and were slightly lower in the intervention arm. | Decrease* | Reduction in sporozoite positive *An. gambiae* s.l., between years (logistic regression *P* = 0.039). |
| Poche *et al.* [50] | D | *An. gambiae* s.s**, An. arabiensis*,*  *An. funestus* | *n/a* | Both *An. arabiensis* and *An. gambiae* s.s reduced significantly 4 weeks post treatment, in at least one site. No statistical analysis for before and after stated for *An. funestus.* | Increase* | *An. gambiae* s.s increased in sporozoite rate, significantly in site 1.  *An. funestus* also increased, but not significantly. |
| Russell *et al.* [53] | D | *An. gambiae* s.s**, An. arabiensis** | *n/a* | Bites per person per night reduced significantly amongst *An. gambiae* complex and *An. funestus* (*P* < 0.05), after introduction of intervention*.* | Decrease* | 18-fold reduction in EIR, in protected versus unprotected person  4.6-fold reduction with high-bed net (with long-lasting insecticide application) coverage versus untreated nets.  Sporozoite prevalence reduced significantly amongst *An. gambiae* complex and *An. funestus* (*P* < 0.05)*.* |
| Russell *et al.* [54] | ID | *An. gambiae* s.s.* | *An arabiensis** | The longitudinal shift in sibling species composition (based on densities) towards *An. arabiensis* was statistically associated with year (β = -1.152, se = 0.038, *P* < 0.0001), but was not related to rainfall patterns (β = -3.079 x 10^-4^, se = 2.04410^-4^, *P* = 0.132). | n/a | n/a |
| Sharp *et al.* [55] *(carbamate data observations only)* | D | *An. melas*, An. funestus*, An gambiae* | *n/a* | *An. funestus* and *An. melas* showed ‘significant reductions’, after second round of IRS (with carbamate). No further explanation of analysis given. | Decrease | No transmission index could be calculated, as all mosquitoes were negative for sporozoites after second spray round. |
| Smith and Draper [56] | D | *An. gambiae*, An. funestus** | *n/a* | There was a statistically significant difference between mosquito densities as determined by one-way ANOVA (*F*_(3,30)_ = 5.770, *p* = 0.003. An LSD Post hoc comparison revealed that densities of *An. funestus* in huts before dieldrin was sprayed, were significantly higher than after (*P* < 0.01), and densities of *An. gambiae* in huts before dieldrin was sprayed, were significantly higher than after spraying (*P* < 0.01). | - | - |
| Sougoufara *et al.* [58] | ID | *An. coluzzii*,*  *An. gambiae** | *An. arabiensis** | The number of bites recorded varied significantly according to species (GLM quasi-Poisson family: ꭓ^2^ = 9.597, *df* = 2, *P* < 0.01) and year (GLM quasi-Poisson family: ꭓ^2^ = 37.725, *df* = 2, *P* < 0.001).  The species x year interaction was also significant (GLM quasi-Poisson family: ꭓ^2^ = 45.869, *df* = 4, *P* < 0.001). | A decrease of 36.8% in 2006 to 12.3% in 2008 in children agead 0-14 years, and from 27.6% in 2006 to 9.0% in 2008 in people aged 15 years or older.  Incidence of malaria attacks in the community decreased 57-fold between 2000 and 2012. | n/a |
| Sougoufara *et al.* [59] | ID | *An. funestus** | *An. gambiae* s.l*.** | According to the GLM analysis, species, period, site of collection, rain and month all had a significant effect on the number of bites. Species x period x month interaction was significant, i.e. the effect of months differ by period but differently for *An. gambiae* s.l*.* and *An. funestus* s.l*.* | Decrease* | The infection rate varied significantly depending on the periods of the study (ꭓ^2^ = 10.648, *P* = 0.014). In P1, a bed net user would have received 40.4 infected bites per year. In p4, a bed net user was expected to encounter 3.2 infected bites per year. |
| Zhou *et al.* [62] | D | *An. funestus*, An. gambiae** | *n/a* | *An. gambiae* density in intervention valley reduced by 96.8% (GLM planned comparison, *F*_(1,14)_ = 7.63, *P* = 0.02)  *An. gambiae* density in the uphill area reduced by 51.6%  *An. funestus* density in the intervention valley reduced by 85.3% (GLM planned comparison *F*_(1,14)_ = 9.16, *P* = 0.01)  *An. funestus* density in the uphill area reduced by 69.2%. | Decrease* | Parasite prevalence in the intervention valley dropped significantly from 63.6% before intervention to 16.4% after intervention (GLM planned comparison, *F*_(1,24)_ = 309.29, *P* < 0.0001). |
| Zhou *et al.* [63] | ID | *An. gambiae** | *An. arabiensis** | Analysis of species composition (based on densities) illustrated that the proportion of *An. arabiensis* rose significantly in Kombewa from 1.7% in 2003 to 61.7% in 2009. (ꭓ^2^ = 65.8, *df* = 1, *P*<0.0001), but decreased significantly to 11.5% in 2010 (compared with 2009, ꭓ^2^ = 119.5, *df* = 1, *P* = 0.0001).  Species composition (based on densities) change in Iguhu was characterised by a significant increase in the proportion of *An. arabiensis* from <1% in 2003 to 18.8% in 2006 (Fisher exact test *P* = 0.001), then by a gradual declining trend from 2006 – 2010 (9.2%. Fisher exact test *P*<0.05). | **Kombewa**  Increase*  **Iguhu**  Decrease* | **Kombewa**  Parasite prevalence decreased slightly from 2003 to 2006 (average 52.8%, range 37-78%), then declined sharply (average 8.9%, range 0-25%) during the last half of 2006 (Tukey-Kramer HSD test, *P*<0.0001). Thereafter it gradually increased throughout 2007 (average 30%) and well into 2008, reaching a monthly rate of 49.6% in 2008, about the same prevalence observed before 2006, and significantly higher than 2007 (Tukey-Kramer HSD test, P<0.05).  **Iguhu**  Decreasing trend in parasite prevalence was observed from 2002 with a notable decline in 2005, however a sharp declining trend occurred after 2006. The monthly parasite prevalence dropped from an average of 33.8% (range from 18-57%) before July 2006 to 7.5% (range from 2-16%) between July 2006 and December 2008 (Tukey-Kramer HSD test of ANOVA with repeated measure, *P*<0.0001). Monthly mean parasite prevalence was 13.0% in 2009, which significantly exceeded the 2007/2008 level. |
| Zhou *et al.* [64] | ID | *An. gambiae* s.l*.** | *An. funestus** | *An. gambiae* s.l*.* differs by being significantly lower in 2008 than 2003 in all sites (no further statistical information provided).  *An. funestus* has rebounded significantly in 2008 across all sites, compared to 2003 (unequal variance *t*-test, *t* = 6.10, *df* = 7, *P* < 0.001). | Decrease* | Parasite prevalence in school children decreased sharply at all sites from 2003-2008 (ꭓ^2^ tests, *P* < 0.01 at all sites). |

**Table S3. Studies without statistically significant changes in absolute densities of vector species.** Any statistical differences were identified from raw data analysis in each of the studies selected for detailed analysis of evidence for release from competition of vectors after suppression of *Anopheles* vector by insecticide-based intervention. *indicates statistically significant result.

| Study | Category of density change | Mosquito population density decreased | Mosquito population density  increased | Mosquito density statistics | | Malaria transmission | | Malaria transmission statistics | |
| --- | --- | --- | --- | --- | --- | --- | --- | --- | --- |
| Abong’o *et al.* [2] | ID | *An. funestus** | *An. arabiensis* | | A statistically significant difference-of-differences was observed between period of mosquito collection and intervention status post-IRS indicating a stronger decline of *An. funestus* in the IRS sites compared to the non-IRS sites (RR = 0.06, 95% CI:0.03-0.13, *P*<0.001).  The mean numbers of *An. arabiensis* collected in indoor CDC-LTs in both interventions and non-intervention sites increased in the post-IRS compared to pre-IRS period, with a statistically different increase only for the non-IRS sites (IRS sites: RR = 1.39. 95% CI:0.78-2.47, *P* = 0.266; non-IRS sites: RR = 3.06. 95% CI 1.59-5.92, *P* = 0.001).  *Anopheles funestus* comprised over 80% of the total *Anopheles* collected in both intervention and non-intervention sites before IRS. While *An. funestus* remained dominant in non-intervention sites after IRS, *An. arabiensis* formed the bulk of all *Anopheles* collected in the intervention sites after IRS. | | Sporozoite infection rates in both *An. funestus* and *An. arabiensis* decreased post IRS. | | n/a |
| Bamou *et al.* [6] | ID | *An. moucheti** | *An. marshalli, An paludism, An. ziemannii* | | In Olama, a significant decrease in mosquito densities was recorded (GLM, Wald ꭓ^2^ = 16.27, *P* = 0.0001).  The increase in density of mosquitoes from which proportions of mosquitoes were determined, was not significant (GLM, Wald ꭓ^2^ = 0.18, *P* = 0.66). | | A decrease in EIR was recorded in both Olama (92%) and Nyabessan (26%), between 2000 and 2016. | | n/a |
|  |  |  |  | |  | |  | |  |
| Bekele *et al.* [8] | D | *n/a* | *An. gambiae* s.l*., An. pharoensis, An. coustani* | | No statistical analysis provided | | Decrease* | | The *Plasmodium* prevalence was decreased in the kebeles that were covered with ITN+IRS as opposed either only ITN or no intervention (*P* < 0.05). |
| Chanda *et al.* [11] *(ITN data observations only)* | D | *An. funestus, An. arabiensis* |  | | No statistical difference between figures | | No vectors tested positive for sporozoites | | n/a |
| Chanda *et al.* [11] *(IRS data observations only)* | I | *n/a* | *An. gambiae* s.l*., An. arabiensis* | | No statistical difference between figures | | No vectors tested positive for sporozoites | | n/a |
| Fontaine *et al.* [15] | D | *n/a* | *An. funestus, An. gambiae* | | *An. funestus* man biting rate reduced from 1.6 bmn to 0.004 bmn  *An. gambiae* 4.6 bmn to 0.01bmn.  No statistical tests undertaken. | | n/a | | n/a |
| Lozano Fuentes *et al.* [27] | ID | *An. arabiensis* | *An. gambiae* s.s**, An. funestus* s.s | | Before/after data used from graphs, indicating confidence intervals.  Treatment vs. control data not used, due to both areas being within 0.5km of one another. | | n/a | | n/a |
| Majori *et al.* [29] | D | *n/a* | *An. gambiae* s.l*.*, An. funestus* | | *An. gambiae* s.l*.* was reduced from 137.09±59.64 females/room in the pre-treatment catch to 0 after treatment, this reduction was significant (*F* = 6.01, *df*_1_=2, *df*_2_=52. *P*<0.01).  A similar reduction was reported for *An. funestus,* though a statistical difference was not mentioned. | | n/a | | n/a |
| Mmbando *et al.*  [34] *(outdoor data observations only)* | D | *An. gambiae*,* species of *Culex* | *An. funestus, Mansonia* spp. | | All *P* values represent IRR of % protection reductions of number of host-seeking mosquitoes attempting to bite volunteers outdoors in local households with push-pull, versus controls:  *An. gambiae* complex 0.49, *P*<0.005  *An. funestus* group -0.48, *P*>0.05  *Mansonia* spp. -3.2, *P*>0.05  *Culex* spp. 0.02, *P*>0.05. | | n/a | | n/a |
| Njan Nloga *et al.* [41] | ID | *An. moucheti, Mansonia* spp. | *Cx. quinquefasciatus* | | ‘Slight’ increase in *Cx. quinquefasciatus* after eight months of introduction of bednets is mentioned, but no statistical analysis performed. | | Decrease* | | Prevalence of malaria parasites was reduced significantly by 40.3% (*Z* = 4.54), in subjects less than 15 years after the installation of bednets |
| Pant *et al.* [47] | ID | *An. gambiae* | *An. funestus* | | n/a | | n/a | | n/a |
| Pant *et al.* [48] | D | *n/a* | *An. gambiae, An. funestus* | | No statistical testing undertaken | | n/a | | n/a |
| Protopopoff *et al.* [51] | D | *n/a* | *An. gambiae* s.s*.*, An. arabiensis, Cx.* spp. | | No significant reduction for *An arabiensis* or *Culex* spp. However, *An. gambiae* s.s density was 85% lower (adjusted IRR 0.15, 95% CI: 0.05-0.44, *P* = 0.001), in the IRS+ITN arm compared to ITN only arm. | | Decrease | | 0.73 OR (95% CI 0.21-2.54), P=0.607 for Sporozoite rate |
| Sharp *et al.* [55] *(pyrethroid data observations only)* | ID | *An. melas*, An. funestus** | *An. gambiae* | | Numbers taken from longitudinal data, after first round of IRS. No statistical analysis explained in text, apart from *An. melas* and *An. funestus* showed ‘significant reductions’. | | Decrease | | After first spray round, sporozoite prevalence reduced from 6.0%, 8.3% and 4.0% for *An. gambiae* s.s*, An. melas* and *An. funestus* to1.8%, 3.1% and 2.3% respectively. No statistical analysis information provided. |
| Trape *et al.* [60] | ID | *An. funestus* | *An. gambiae* s.l*.* | | *An. funestus* mean monthly human biting rate decreased substantially after the introduction of LLINs, but the mean monthly human biting rate for *An. gambiae* increased. Data taken from graph. No statistical analysis available for time points. | | Decrease, and then increase | | Initial decrease between December 2010 and August 2008 (*P*=0.0001 by two-sided binomial exact test).  Increase between January 2007 and July 2010 (*P*<0.0001 by Fisher exact test). |

**Category ID studies dismissed from qualitative assessment in this review, as providing only weak evidence for competitive release or suggesting alternative explanations for changes in vector species composition**

***Marginal changes in population densities***

Mmbando et al. [34] examined the entomological effects of a push-pull system, where mosquitoes are both repelled from human hosts by dispensers containing transfluthrin and attracted to lethal odour-baited landing boxes in Tanzania. While the authors reported decreases in the densities of *An. gambiae* s.l. and species of *Culex* with concomitant increases in the densities of *An. funestus* and species of *Mansonia*, these differences were relative low, rendering results inconclusive.

Oloo et al. [44] examined the impact on population densities of *An. gambiae* s.l. and *An. funestus* between June 1991 and June 1992 following the introduction in May 1991 of permethrin impregnated sisal curtains. They found indoor biting rates for both *An. gambiae* s.l. and *An. funestus* were substantially reduced in the intervention village compared with control sites. However, there was an increase in biting rates for *An. funestus* compared to controls between April and July 1992 that was not observed in *An. gambiae*. However, the duration of the study was too short to provide any data beyond June 1992 after one year of investigation.

***Insecticide resistance***

Helinski et al*.* [21] examined the entomological impacts of LLIN universal coverage in March/April at four sites in Midwestern Uganda, of which for Buliisa only, they recorded density data for both *An. gambiae* s.l. and *An. funestus*. In Buliisa, light trap collection (LTC) data revealed that 66% of all samples were *An. funestus*, of which 97% were *An. funestus* s.s*.*, 21% of samples were *An. gambiae* s.s., and 13% *An. arabiensis*. By May 2011, the density of *An. gambiae* s.l. had increased significantly from pre-intervention levels while *An. funestus* had almost completely disappeared. Unfortunately, the relative proportions of *An. gambiae* s.s. and *An. arabiensis* over these different time points were not reported. *An. funestus* resurged over 2012 so that it again became the dominant species. The authors tested for the *kdr* L1014S mutations in *An. gambiae* s.l. samples and found that its frequency increased from 72% of *An. gambiae* s.s. in 2009 to 87, 96 and 86% in 2010, 2011 and 2012, respectively, although phenotypic resistance was not assessed. By contrast, no samples of *An. arabiensis* were positive for the *kdr* mutation over this period. Thus, the increase in density of *An. gambiae* s.l. in 2011 could have been caused by increases in numbers of insecticide resistant *An. gambiae* s.s*.*, or by increases in numbers of *An. arabiensis* caused by changes in inter-species competitive interactions, or by a combination of the former and latter. A possible explanation for the resurgence in *An. funestus* was the emergence of insecticide resistance in that species, although neither its *kdr* status nor phenotypic resistance was tested in the study. The rebound of *An. funestus* may also have been caused by the increased density of a species other than *An. funestus* s.s. from the group. It is also worth noting that resistance in a species would be expected to maintain the density of a population in the face of insecticide use, but not to yield an increase in density unless there was additionally some release from competition on the resistant species.

Zhou et al. [64] investigated the impact of mass distribution of ITNs in western Kenya between 2006 and 2015. The percent of ITN usage increased in all villages from averages of 23.3 ± 1.1, 43.8 ± 8.4, and 85.6 ± 14.0 in 2006, 2010 and 2011, respectively. In 2006, *An. gambiae* s.l*.* was the predominant vector in Iguhu and Marani, and *An. funestus* in Kombewa. *An. gambiae* s.l. and *An. funestus* decreased in density in all villages between 2006 and 2008, but then increased in 2011 and 2015. *An. gambiae* s.l. became the dominant vector in Kombewa, and *An. funestus* in Marani. In Marani, for both vectors, there were greater densities in the ITN arm of study, compared to non-ITN arm. However, these effects appear to have been mediated by pyrethroid resistance in *An. gambiae* [65, 66] and, as suggested by the authors, in *An. funestus*. Even though these increases in densities occurred parallel to increases in bednet uptake, malaria transmission reduced over the period of the study.

***Rainfall***

Sougoufara et al. [59] investigated the impact of the introduction and two rounds of renewal of LLINs in Dielmo, Senegal over four different time points. P1 was during the pre-intervention period between July 2006 and June 2008. P2 was after the first implementation of LLINs between July 2008 and June 2011. P3 was after the first renewal of LLINs between July 2011 and June 2014. P4 was after the second renewal of LLINs between August 2014 and April 2016. The density of *An. funestus* rapidly declined following the initial introduction of LLINs and remained low at 12.5, 1.0, 1-2, and 1-2 bpn in P1, P2, P3, and P4, respectively. By contrast, the density of *An. gambiae* s.l. initially increased following introduction of LLINs but subsequently decreased following the second renewal of LLINs, with 8.7, 12.3, 4.8, and 1.7 bpn in P1, P2, P3, and P4, respectively. Of note, before the intervention, *An. funestus* was the dominant species involved in year-round malaria transmission, whereas the prevalence of *An. gambiae* was associated with the rainy season.

During the pre-intervention period there were two years with lower than normal rainfall which reduced water resources required for larval habitats so that *An. funestus* numbers had already started to reduce during P1 between 2006 and 2008 and continued to decline post-intervention. By contrast, the increase in density of *An. gambiae* s.l. in P2 was associated with increases in rainfall between 2008 and 2010. It was only following renewal of LLINs that *An. gambiae* s.l. declined in P3 between July 2011 and June 2014. Thus, the authors proposed that the increase of *An. gambiae* s.l. could be explained by the increase in rainfall as a paramount resource required for the development of larval habitats and numbers of adult *An. gambiae* s.l.

**Supplementary references**

1. WHO. Global Partnership to Roll Back Malaria. (‎2000)‎. The African summit on Roll Back Malaria, Abuja, Nigeria, April 25 2000. World Health Organization.

2. Abong'o B, Gimnig JE, Torr SJ, Longman B, Omoke D, Muchoki M, et al. Impact of indoor residual spraying with pirimiphos-methyl (Actellic 300CS) on entomological indicators of transmission and malaria case burden in Migori County, western Kenya. Sci Rep. 2020;10:4518.

3. Akono PN, Tcheugoue GRJ, Mbida JA, Tonga C, Lehman LG. Higher mosquito aggressiveness and malaria transmission following the distribution of alpha-cypermethrin impregnated mosquito nets in a district of Douala, Cameroon. Afr Entomol. 2018;26 2:429-36.

4. Alegana VA, Kigozi SP, Nankabirwa J, Arinaitwe E, Kigozi R, Mawejje H, et al. Spatio-temporal analysis of malaria vector density from baseline through intervention in a high transmission setting. Parasit Vectors. 2016;9:637.

5. Antonio-Nkondjio C, Demanou M, Etang J, Bouchite B. Impact of cyfluthrin (Solfac EW050) impregnated bed nets on malaria transmission in the city of Mbandjock : lessons for the nationwide distribution of long-lasting insecticidal nets (LLINs) in Cameroon. Parasit Vectors. 2013;6:10.

6. Bamou R, Mbakop LR, Kopya E, Ndo C, Awono-Ambene P, Tchuinkam T, et al. Changes in malaria vector bionomics and transmission patterns in the equatorial forest region of Cameroon between 2000 and 2017. Parasit Vectors. 2018;11:464.

7. Bayoh MN, Mathias DK, Odiere MR, Mutuku FM, Kamau L, Gimnig JE, et al. *Anopheles gambiae*: historical population decline associated with regional distribution of insecticide-treated bed nets in western Nyanza Province, Kenya. Malar J. 2010;9:62.

8. Bekele D, Belyhun Y, Petros B, Deressa W. Assessment of the effect of insecticide-treated nets and indoor residual spraying for malaria control in three rural kebeles of Adami Tulu District, South Central Ethiopia. Malar J. 2012;11:127.

9. Bogh C, Pedersen EM, Mukoko DA, Ouma JH. Permethrin-impregnated bednet effects on resting and feeding behaviour of lymphatic filariasis vector mosquitoes in Kenya. Med Vet Entomol. 1998;12:52-9.

10. Bukhari T, Takken W, Githeko AK, Koenraadt CJ. Efficacy of aquatain, a monomolecular film, for the control of malaria vectors in rice paddies. PLoS One. 2011;6 6:e21713.

11. Chanda E, Coleman M, Kleinschmidt I, Hemingway J, Hamainza B, Masaninga F, et al. Impact assessment of malaria vector control using routine surveillance data in Zambia: implications for monitoring and evaluation. Malar J. 2012;11:437.

12. Coleman S, Dadzie SK, Seyoum A, Yihdego Y, Mumba P, Dengela D, et al. A reduction in malaria transmission intensity in Northern Ghana after 7 years of indoor residual spraying. Malar J. 2017;16:324.

13. Dabire RK, Diabate A, Baldet T, Pare-Toe L, Guiguemde RT, Ouedraogo JB, et al. Personal protection of long lasting insecticide-treated nets in areas of *Anopheles gambiae* s.s. resistance to pyrethroids. Malar J. 2006;5:12.

14. Degefa T, Yewhalaw D, Zhou G, Lee MC, Atieli H, Githeko AK, et al. Indoor and outdoor malaria vector surveillance in western Kenya: implications for better understanding of residual transmission. Malar J. 2017;16:443.

15. Fontaine RE, Joshi GP, Pradhan GD. Entomological evaluation of fenitrothion (OMS-43) as a residual spray for the control of an *An. gambiae* and *An. funestus*, Kisumu, Kenya. World Health Organization, Geneva. 1975.

16. Futami K, Dida GO, Sonye GO, Lutiali PA, Mwania MS, Wagalla S, et al. Impacts of insecticide treated bed nets on *Anopheles gambiae* s.l. populations in Mbita district and Suba district, Western Kenya. Parasit Vectors. 2014;7:63.

17. Gillies MT, Smith A. The effect of a residual house-spraying campaign in East Africa on species balance in the *Anopheles funestus* group. The replacement of *A. funestus* Giles by *A. rivulorum* Leeson. Bull Entomol Res. 1960;51;243-52.

18. Gimnig JE, Vulule JM, Lo TQ, Kamau L, Kolczak MS, Phillips-Howard PA, et al. Impact of permethrin-treated bed nets on entomologic indices in an area of intense year-round malaria transmission. Am J Trop Med Hyg. 2003;68:16-22.

19. Gimnig JE, Kolczak MS, Hightower AW, Vulule JM, Schoute E, Kamau L, et al. Effect of permethrin-treated bed nets on the spatial distribution of malaria vectors in western Kenya. Am J Trop Med Hyg. 2003;68:115-20.

20. Govella NJ, Ogoma SB, Paliga J, Chaki PP, Killeen G. Impregnating hessian strips with the volatile pyrethroid transfluthrin prevents outdoor exposure to vectors of malaria and lymphatic filariasis in urban Dar es Salaam, Tanzania. Parasit Vectors. 2015;8:322.

21. Helinski ME, Nuwa A, Protopopoff N, Feldman M, Ojuka P, Oguttu DW, et al. Entomological surveillance following a long-lasting insecticidal net universal coverage campaign in Midwestern Uganda. Parasit Vectors. 2015;8:458.

22. Kapesa A, Kweka EJ, Atieli H, Kamugisha E, Zhou G, Githeko AK, et al. Why some sites are responding better to anti-malarial interventions? A case study from western Kenya. Malar J. 2017;16:498.

23. Kenea O, Balkew M, Tekie H, Deressa W, Loha E, Lindtjorn B, et al. Impact of combining indoor residual spraying and long-lasting insecticidal nets on *Anopheles arabiensis* in Ethiopia: results from a cluster randomized controlled trial. Malar J. 2019;18:182.

24. Kweka EJ, Lee MC, Mwang'onde BJ, Tenu F, Munga S, Kimaro EE, et al. Bio-efficacy of deltamethrin based durable wall lining against wild populations of *Anopheles gambiae* s.l. in Northern Tanzania. BMC Res Notes. 2017;10:92.

25. Labbo R, Czeher C, Djibrila A, Arzika I, Jeanne I, Duchemin JB. Longitudinal follow-up of malaria transmission dynamics in two villages in a Sahelian area of Niger during a nationwide insecticide-treated bednet distribution programme. Med Vet Entomol. 2012;26:386-95.

26. Lindblade KA, Gimnig JE, Kamau L, Hawley WA, Odhiambo F, Olang G, et al. Impact of sustained use of insecticide-treated bednets on malaria vector species distribution and Culicine mosquitoes. J Med Entomol. 2006;43:428-32.

27. Lozano-Fuentes S, Kading RC, Hartman DA, Okoth E, Githaka N, Nene V, et al. Evaluation of a topical formulation of eprinomectin against *Anopheles arabiensis* when administered to Zebu cattle (*Bos indicus*) under field conditions. Malar J. 2016;15:324.

28. Mahande AM, Mosha FW, Mahande JM, Kweka EJ. Role of cattle treated with deltamethrine in areas with a high population of Anopheles arabiensis in Moshi, Northern Tanzania. Malar J. 2007;6:109.

29. Majori G, Sabatinelli G, Coluzzi M. Efficacy of permethrin-impregnated curtains for malaria vector control. Med Vet Entomol. 1987;1:185-92.

30. Mbogo CN, Baya NM, Ofulla AV, Githure JI, Snow RW. The impact of permethrin-impregnated bednets on malaria vectors of the Kenyan coast. Med Vet Entomol. 1996;10:251-9.

31. McCann RS, Messina JP, MacFarlane DW, Bayoh MN, Gimnig JE, Giorgi E, et al. Explaining variation in adult *Anophele*s indoor resting abundance: the relative effects of larval habitat proximity and insecticide-treated bed net use. Malar J. 2017;16 1:288.

32. Meyers JI, Pathikonda S, Popkin-Hall ZR, Medeiros MC, Fuseini G, Matias A, et al. Increasing outdoor host-seeking in *Anopheles gambiae* over 6 years of vector control on Bioko Island. Malar J. 2016;15:239.

33. Meyrowitsch DW, Pedersen EM, Alifrangis M, Scheike TH, Malecela MN, Magesa SM, et al. Is the current decline in malaria burden in sub-Saharan Africa due to a decrease in vector population? Malar J. 2011;10:188.

34. Mmbando AS, Ngowo HS, Kilalangongono M, Abbas S, Matowo NS, Moore SJ, et al. Small-scale field evaluation of push-pull system against early- and outdoor-biting malaria mosquitoes in an area of high pyrethroid resistance in Tanzania. Wellcome Open Res. 2017;2:112.

35. Molineaux L, Shidrawi GR, Clarke JL, Boulzaguet R, Ashkar T, Dietz K. The impact of propoxur on *Anopheles gambiae* s.l. and some other anopheline populations, and its relationship with some pre-spraying variables. Bull World Health Organ. 1976;54:379-89.

36. Musiime AK, Smith DL, Kilama M, Geoffrey O, Kyagamba P, Rek J, et al. Identification and characterization of immature *Anopheles* and culicines (Diptera: Culicidae) at three sites of varying malaria transmission intensities in Uganda. Malar J. 2020;19:221.

37. Mutuku FM, King CH, Mungai P, Mbogo C, Mwangangi J, Muchiri EM, et al. Impact of insecticide-treated bed nets on malaria transmission indices on the south coast of Kenya. Malar J. 2011;10:356.

38. Mwangangi JM, Muturi EJ, Muriu SM, Nzovu J, Midega JT, Mbogo C. The role of *Anopheles arabiensis* and *Anopheles coustani* in indoor and outdoor malaria transmission in Taveta District, Kenya. Parasit Vectors. 2013;6:114.

39. Mwangangi JM, Mbogo CM, Orindi BO, Muturi EJ, Midega JT, Nzovu J, et al. Shifts in malaria vector species composition and transmission dynamics along the Kenyan coast over the past 20 years. Malar J. 2013;12:13.

40. Najera JA, Shidrawi GR, Gibson FD, Stafford JS. A large-scale field trial of malathion as an insecticide for antimalarial work in southern Uganda. B World Health Organ. 1967;36:913-35.

41. Njan Nloga AM, Messi J, Carnevale P, URL:. Efficacy of lambdacyalothrin impregnated bednets against mosquitoes with particular reference to *Anopheles moucheti* at Ebogo, Cameroon. Int J Trop Med. 2006;1:71-6.

42. Njoroge MM, Tirados I, Lindsay SW, Vale GA, Torr SJ, Fillinger U. Exploring the potential of using cattle for malaria vector surveillance and control: a pilot study in western Kenya. Parasit Vectors. 2017;10:18.

43. Odhiambo MT, Skovmand O, Vulule JM, Kokwaro ED. Evaluation of polyethylene-based long lasting treated bed net netprotect on *Anopheles* mosquitoes, malaria incidence, and net longivity in Western kenya. J Trop Med. 2013;2013:563957.

44. Oloo A, Githeko A, Adungo N, Karanja D, Vulule J, Kisia-Abok I, et al. Field trial of permethrin impregnated sisal curtains in malaria control in western Kenya. East Afr Med J. 1996;73:735-40.

45. Osse R, Aikpon R, Padonou GG, Oussou O, Yadouleton A, Akogbeto M. Evaluation of the efficacy of bendiocarb in indoor residual spraying against pyrethroid resistant malaria vectors in Benin: results of the third campaign. Parasit Vectors. 2012;5:163.

46. Ouattara AF, Dagnogo M, Constant EA, Kone M, Raso G, Tanner M, et al. Transmission of malaria in relation to distribution and coverage of long-lasting insecticidal nets in central Cote d'Ivoire. Malar J. 2014;13:109.

47. Pant CP, Rosen P, Joshi GP, Pearson JA, Ramasamy M, Renaud P, et al. A village-scale trial of OMS-708 (Mobam) for the control of *Anopheles gambiae* and *Anopheles funestus* in northern Nigeria. Bull World Health Organ. 1969;41:316-9.

48. Pant CP, Joshi GP, Rosen P, Pearson JA, Renaud P, Ramasamy M, et al. A village-scale trial of OMS-214 (Dicapthon) for the control of *Anopheles gambiae* and *Anopheles funestus* in northern Nigeria. Bull World Health Organ. 1969;41:311-5.

49. Pinder M, Jawara M, Jarju LBS, Salami K, Jeffries D, Adiamoh M, et al. Efficacy of indoor residual spraying with dichlorodiphenyltrichloroethane against malaria in Gambian communities with high usage of long-lasting insecticidal mosquito nets: a cluster-randomised controlled trial. Lancet. 2015;385:1436-46.

50. Poche RM, Githaka N, van Gool F, Kading RC, Hartman D, Polyakova L, et al. Preliminary efficacy investigations of oral fipronil against *Anopheles arabiensis* when administered to Zebu cattle (*Bos indicus*) under field conditions. Acta Trop. 2017;176:126-33.

51. Protopopoff N, Wright A, West PA, Tigererwa R, Mosha FW, Kisinza W, et al. Combination of insecticide treated nets and indoor residual spraying in Northern Tanzania provides additional reduction in vector population density and malaria transmission rates compared to insecticide treated nets alone: a randomised control trial. PLoS One. 2015;10:e0142671.

52. Ratovonjato J, Randrianarivelojosia M, Rakotondrainibe ME, Raharimanga V, Andrianaivolambo L, Le Goff G, et al. Entomological and parasitological impacts of indoor residual spraying with DDT, alphacypermethrin and deltamethrin in the western foothill area of Madagascar. Malar J. 2014;13:21.

53. Russell TL, Lwetoijera DW, Maliti D, Chipwaza B, Kihonda J, Charlwood JD, et al. Impact of promoting longer-lasting insecticide treatment of bed nets upon malaria transmission in a rural Tanzanian setting with pre-existing high coverage of untreated nets. Malar J. 2010;9:187.

54. Russell TL, Govella NJ, Azizi S, Drakeley CJ, Kachur SP, Killeen GF. Increased proportions of outdoor feeding among residual malaria vector populations following increased use of insecticide-treated nets in rural Tanzania. Malar J. 2011;10:80.

55. Sharp B, Kleinschmidt I, Streat E, Maharaj R, Barnes KG, Durrheim DN, et al. Seven years of regional malaria control collaboration - Mozambique, South Africa, and Swaziland. Am J Trop Med Hyg. 2007;76:42-7.

56. Smith A, Draper CC. Malaria in the Taveta area of Kenya and Tanganyika. Part II. Results after three and a half years' treatment of huts with dieldrin. East Afr Med J. 1959;36:629-43.

57. Smith A. Malaria in the Taveta area of Kenya and Tanzania. IV. Entomological findings six years after the spraying period. East Afr Med J. 1966;43:7-18.

58. Sougoufara S, Harry M, Doucoure S, Sembene PM, Sokhna C. Shift in species composition in the *Anopheles gambiae* complex after implementation of long-lasting insecticidal nets in Dielmo, Senegal. Med Vet Entomol. 2016;30:365-8.

59. Sougoufara S, Thiaw O, Cailleau A, Diagne N, Harry M, Bouganali C, et al. The Impact of periodic distribution campaigns of long-lasting insecticidal-treated bed nets on malaria Vector dynamics and human exposure in Dielmo, Senegal. Am J Trop Med Hyg. 2018;98:1343-52.

60. Trape J-F, Tall A, Diagne N, Ndiath O, Ly AB, Faye J, et al. Malaria morbidity and pyrethroid resistance after the introduction of insecticide-treated bednets and artemisinin-based combination therapies: a longitudinal study. Lancet Infect Dis. 2011;11:925-32.

61. Wragge SE, Toure D, Coetzee M, Gilbert A, Christian R, Segoea G, et al. Malaria control at a gold mine in Sadiola District, Mali, and impact on transmission over 10 years. Trans R Soc Trop Med Hyg. 2015;109:755-62.

62. Zhou G, Githeko AK, Minakawa N, Yan G. Community-wide benefits of targeted indoor residual spray for malaria control in the western Kenya highland. Malar J. 2010;9:67.

63. Zhou G, Afrane YA, Vardo-Zalik AM, Atieli H, Zhong D, Wamae P, et al. Changing patterns of malaria epidemiology between 2002 and 2010 in Western Kenya: the fall and rise of malaria. PLoS One. 2011;6:e20318.

64. Zhou G, Lee MC, Githeko AK, Atieli HE, Yan G. Insecticide-treated net campaign and malaria transmission in Western Kenya: 2003-2015. Front Public Health. 2016;4:153.

65. Strode C, Donegan S, Garner P, Enayati AA, Hemingway J. The impact of pyrethroid resistance on the efficacy of insecticide-treated bed nets against African anopheline mosquitoes: systematic review and meta-analysis. PLoS Med. 2014;11:e1001619.

66. Wanjala CL, Zhou G, Mbugi J, Simbauni J, Afrane YA, Ototo E, et al. Insecticidal decay effects of long-lasting insecticide nets and indoor residual spraying on Anopheles gambiae and Anopheles arabiensis in Western Kenya. Parasit Vectors. 2015;8:588.
